# Galanin and Adrenomedullin Plasma Responses During Artificial Gravity on a Human Short-Arm Centrifuge

**DOI:** 10.3389/fphys.2018.01956

**Published:** 2019-02-01

**Authors:** Julia Winter, Charles Laing, Bernd Johannes, Edwin Mulder, Bianca Brix, Andreas Roessler, Johannes Reichmuth, Joern Rittweger, Nandu Goswami

**Affiliations:** ^1^Department of Aerospace Physiology, Institute for Aerospace Medicine, German Aerospace Center e.V. (DLR), Cologne, Germany; ^2^Centre for Human and Aerospace Physiological Sciences, King’s College London, London, United Kingdom; ^3^Gravitational Physiology and Medical Research Unit, Physiology Division, Otto Loewi Center for Research in Vascular Biology, Immunity, and Inflammation, Medical University of Graz, Graz, Austria; ^4^Department of Pediatrics and Adolescent Medicine, University of Cologne, Cologne, Germany

**Keywords:** syncope, orthostatic intolerance, gravity gradient, post-flight orthostatic intolerance, human spaceflight

## Abstract

Galanin and adrenomedullin plasma responses to head-up tilt and lower body negative pressure have been studied previously. However, to what extent short-arm human centrifugation (SAHC) affects these responses is not known. In this study, we assessed how the application of variable gradients of accelerations (*ΔG_z_*) via shifting of the rotation axis during centrifugation affects selected hormonal responses. Specifically, we tested the hypothesis, that *cardiovascular modulating hormones such as galanin and adrenomedullin will be higher in non-finishers* (participants in whom at least one of the pre-defined criteria for presyncope was fulfilled) *when compared to finishers* (participants who completed the entire protocol in both sessions) *during SAHC exposure.* Twenty healthy subjects (10 women and 10 men) were exposed to two g-levels [1 G_z_ and 2.4 G_z_ at the feet (G_z_Feet_)] in two positions (axis of rotation placed above the head and axis of rotation placed at the heart level). Elevated baseline levels of galanin appeared to predict orthostatic tolerance (*p* = 0.054) and seemed to support good orthostatic tolerance during 1 G_z_Feet_ SAHC (*p* = 0.034). In finishers, 2.4 G_z_Feet_ SAHC was associated with increased galanin levels after centrifugation (*p* = 0.007). For adrenomedullin, the hypothesized increases were observed after centrifugation at 1 G_z_Feet_ (*p* = 0.031), but not at 2.4 G_z_Feet_, suggesting that other central mechanisms than local distribution of adrenomedullin predominate when coping with central hypovolemia induced by SAHC (*p* > 0.14). In conclusion, baseline galanin levels could potentially be used to predict development of presyncope in subjects. Furthermore, galanin levels increase during elevated levels of central hypovolemia and galanin responses appear to be important for coping with such challenges. Adrenomedullin release depends on degree of central hypovolemia induced fluid shifts and a subject’s ability to cope with such challenges. Our results suggest that the gradient of acceleration (*ΔG_z_*) is an innovative approach to quantify the grade of central hypovolemia and to assess neurohormonal responses in those that can tolerate (*finishers*) or not tolerate (*non-finishers*) artificial gravity (AG). As AG is being considered as a preventing tool for spaceflight induced deconditioning in future missions, understanding effects of AG on hormonal responses in subjects who develop presyncope is important.

## Introduction

Central hypovolemia leads to changes in hemodynamic variables as well as in vasoactive endocrine hormones. These include the classical volume regulating hormones such as renin, angiotensin, aldosterone (RAAS), atrial natriuretic peptide (ANP) and vasopressin. Common stimuli for exploring the effect of central hypovolemia are HUT, LBNP (Goswami et al., 2008, 2019) or HUT in combination with LBNP (Bondanelli et al., 2003; Hinghofer-Szalkay et al., 2011; Goswami et al., 2012; O’shea et al., 2015). Specifically, a rapid elevation of plasma adrenaline and noradrenaline and, after a 10- to 20-min delay, renin-angiotensin system activation, leading to elevated plasma renin activity, angiotensin II and aldosterone is seen (degli Uberti et al., 1996; Convertino et al., 1998; Rossler et al., 1999; Hinghofer-Szalkay et al., 2006, 2011). Arginine vasopressin (AVP), synthesized in the hypothalamus and stored in the posterior pituitary gland, is released in response to hypotension and hypovolemia with some delay (Hirsch et al., 1993; Bichet, 2016). Its main functions include control of water body homeostasis via increases in water reabsorption in the kidney thus increasing the blood pressure (Chopra et al., 2011; Bichet, 2016). Unsurprisingly, AVP plays a key role in the development of orthostatic intolerance: Levels of plasma AVP increase several fold when the endpoint of cardiovascular stability (presyncope) is reached (Hinghofer-Szalkay et al., 2011). For details of the responses RAAS and vasopressin during artificial gravity (AG) application the reader is referred to Yang et al. (2011).

Since the last 20 years, research has now extended to include other hormones that are altered during orthostatic loading and/or central hypovolemia. These include galanin and adrenomedullin (degli Uberti et al., 1996; Bondanelli et al., 2003; Hinghofer-Szalkay et al., 2006, 2011; O’shea et al., 2015). Galanin is a peptide hormone released by the neurohypophysis. Evidence suggests that plasma galanin is increased during orthostatic challenge thus highlighting it’s potential as a marker of presyncope (Hinghofer-Szalkay et al., 2006, 2011; O’shea et al., 2015). Galanin responses have been proposed as reflectors of sympathetic drive (Hinghofer-Szalkay et al., 2006) and appear closely related to heart rate increases (degli Uberti et al., 1996; Bondanelli et al., 2003). Adrenomedullin was first detected in the adrenal gland in human pheochromocytoma but it also seems to originate in endothelial cells (Kitamura et al., 1993; Nishikimi et al., 2001). Acting via paracrine pathways, adrenomedullin exerts rapid, strong and long-lasting vasodilatory effect by increasing NO-production in endothelial cells (Kitamura et al., 1993; Eto, 2001; Ueda et al., 2005). Other studies have reported that changes in plasma adrenomedullin during orthostatic challenge commensurate with the duration and intensity of the central hypovolemia (Rossler et al., 1999; Hinghofer-Szalkay et al., 2011; O’shea et al., 2015). Due to its vasodilatory effects, adrenomedullin is believed to play an important role in the development of presyncope: adrenomedullin increases have been associated with decreases in orthostatic tolerance times (Gajek et al., 2004).

### Short-Arm Human Centrifugation (SAHC): Effects and Benefits

Short-arm human centrifugation (SAHC) offers a novel approach to explore the effect of volume shift upon hormonal control of the cardiovascular system. The gradient acceleration field in SAHC provides a hydrostatic pressure profile that increases quadratically with the distance from the center of rotation, which contrasts with the linear profile in constant 1g-fields. Furthermore, in our study the placement of axis of rotation results in two different pressures of fluid shift to lower extremities and two different grades of central hypovolemia. SAHC is believed to cause central hypovolemia by passive blood shifting toward the lower limbs leading to reduced venous return, cardiac output, and inadequate cerebral perfusion with oxygenated blood (Arthur and Kaye, 2000), which could lead to the development of a presyncopal situation. Presyncope is characterized by symptoms like dizziness, light-headedness, sweating, and results ultimately in transient loss of consciousness (Goswami et al., 2015; Shen et al., 2017).

Short-arm human centrifugation induced centrifugal acceleration, which is imparted in the head-to-toe body axis (*z*-axis), has been proposed as an effective countermeasure against detrimental effects of microgravity (Clément and Bukley, 2007; Clement et al., 2016). Evidence suggests, that exposure to centrifugation, could improve orthostatic tolerance (Goswami et al., 2015). As post-flight orthostatic intolerance occurs commonly when astronauts return from space (Blaber et al., 2011; Goswami et al., 2015; Verma et al., 2016), artificial gravity (AG) has been proposed as a countermeasure against post-spaceflight orthostatic intolerance.

Since RAAS and vasopressin responses during AG application have been previously reported (see Yang et al., 2011), in this study the endocrinological response of galanin and adrenomedullin during varying grades of central hypovolemia induced by SAHC were studied. Specifically, short-term hormonal concentrations to SAHC by application of two different centers of rotation and two different g-levels were investigated in a crossover design in healthy women and men. We tested the hypothesis, *that cardiovascular modulating hormones such as galanin and adrenomedullin were higher in finishers (participants who completed the entire protocol in both sessions) when compared to non-finishers (participants in whom at least one of the pre-defined criteria for presyncope was fulfilled) during SHAC exposure*.

## Materials and Methods

### Design and Setting

The study was conducted in autumn 2014 at the Institute for Aerospace Medicine of the German Center for Aerospace Medicine (DLR) in Cologne, Germany. The protocol (Lfd Nr. 2014123) was approved by the ethics committee of the Aerztekammer Nordrhein (Northern Rhine Medical Council), Duesseldorf, Germany and conformed to the Declaration of Helsinki. Subjects gave their written informed consent before inclusion into the study.

### Subjects

Twenty healthy normotensive female and male subjects participated in the study. Subjects were excluded from study participation if they had practiced drug abuse or, were smokers. Further exclusion criteria were arterial hypertension, diagnosed diabetes mellitus, any muscle or joint disease, herniated disk, chronic back pain, history of epileptic seizures or heart disease or possession of a cardiac pacemaker, or who were pregnant or had histories of orthostatic intolerance.

### Experimental Protocol

The protocol consisted of two visits with five passive spins of centrifugation of 10 min duration during each visit. An 8-week wash-out-period was incorporated between the two visits to avoid training effects. Subjects were centrifuged lying on their back, and with their head orientated toward the axis of rotation.

**FIGURE 1:**
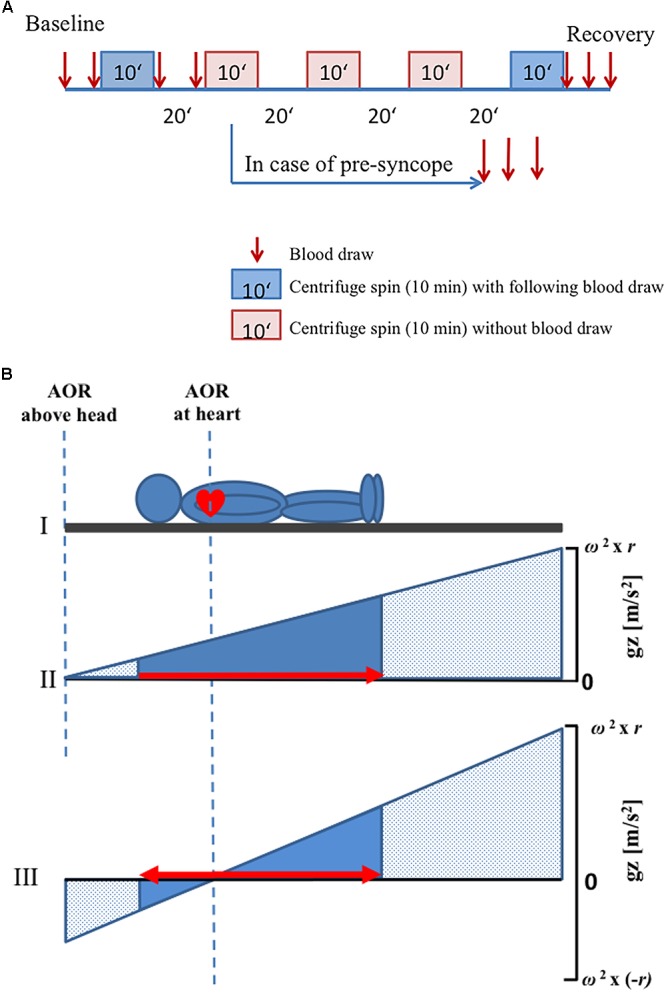
Schematic diagram of the protocol. **(A)** Every visit consisted of five centrifuge spins (10 min duration) with different positions and g-levels. Blood samples were drawn before and after the first and the last centrifuge spin (*red arrows*). A 20-min break was observed between the centrifuge spins. In case of any presyncope, interruption of centrifugation was carried out and additional three blood draws were made. **(B)** Axis of rotation was placed either above the head or at the heart level. ΔG_z_ was computed as the difference in g-level at the top at the head and the g-level at the sole of the feet. In panels (II) and (III), the *x*-axis depicts the location in the body where stress was applied. The area under the curve (*solid blue*) conceptualizes the integrated whole-body-g-stress. The *solid triangles* represent the g-stress which affects to the subject while the *stippled area* represent the (possible) g-Stress along the centrifuge. The *red arrows* indicate the directions of fluid shift. Note that fluid shifts are footward for AOR above the head only (panel II) but that a small gradient pushes the fluid headward, when the AOR is placed at the heart level (panel III). That’s why the gradient of g-level is increased when AOR is placed at the heart level as compared with AOR above the head. AOR, axis of rotation.

After being positioned on the centrifuge, subjects were secured by a harness and instrumented for monitoring of continuous physiological variables (including ECG, pulse oximetry, and beat-to-beat finger blood pressure control). Ten minutes before centrifugation commenced, data recording started for baseline assessment (supine resting period on the centrifuge nacelle), and contiguous centrifugation spins were separated by a 25-min break (including ramp-ups and ramp-downs and position changes). After the second centrifuge spin, there was a 25-min break, during which toilet usage was allowed, and standardized muesli bar and clear water (5 ml per kilogram weight) were provided. After another 10 min of baseline assessment, the subsequent spins took place. At the end, a 20-min recovery phase was included (Figure 1A).

A randomized schedule was used for the applied gradients (1 G_z_ or 2.4 G_z_ at feet (G_z_Feet_), center of rotation placed above the head or at the heart level). A g-level of 2.4 G_z_Feet_ was the greatest possible for the shortest subject (at 38 rpm). Anthropometric parameters of each subject were used to determine the two positions of axis of rotation. Measured specifically was the distance between apex of the heart and vertex of the head, as this distance was used to determine the length of radius that was used during centrifugation in each subject. When the axis of rotation was located above the head position, the distance between axis of rotation and vertex of the head was equal to the distance between the vertex of the head and apex of the heart. The other rotational axis used was located at the apex of the heart (Figure 1B).

Presyncope was defined as a simultaneous blood pressure and heart rate drop for at least 5 seconds with impaired vision, light-headedness or feeling of faintness experienced by the subject [following clinical criteria of the “Guidelines for the diagnosis and management of syncope” of the European Society of Cardiology (version of 2017), Shen et al., 2017]. In case of development of presyncopal signs and symptoms, the centrifugation protocol was immediately terminated by the medical doctor. An occurrence of presyncope during any spin of centrifugation defined a subject as belonging to the group of non-finishers, independent of the visit or spin of centrifugation during which the presyncope occurred. Nevertheless, the subject could continue with the centrifuge protocol. In case of a further presyncope in another spin during the same visit, the procedure of centrifugation was stopped, because no more than two presyncopal events per subject and visit were allowed in this study (Laing et al., 2016).

### Effect of Centrifugation

Centrifugal acceleration (G_z_) is a function of angular velocity (ω) and radius (r): ΔG_z_ = ω^2^ ∗ r. Thus, by manipulation of either of the two variables, at least theoretically, any g-level can be achieved. The gradient of acceleration can be conceptualized as the ΔG_z_-difference between representative points along the *z*-axis of the body, e.g., the head and the foot (Figure 1B). Moreover, the gradient ΔG_z_ depends on both ω and r, thus implying that varying ΔG_z_ will lead to different fluid pressure distributions in the body. In other words, to be meaningful the G_z_ challenges during centrifugation need.

### Blood Sample Protocol

Venous blood was taken from an 18-Gauge cannula placed in the left cubital region. Blood was taken twice during baseline, immediately after first centrifugation and immediately before second centrifugation (i.e., 19 min after stopping the centrifuge). Furthermore, blood was taken three times (immediately, 9 and 19 min after stopping) following the last spin of centrifugation. No blood was sampled in between single spins (Figure 1A). In case of an aborted centrifugation due to presyncope development, additional three blood samples were taken: immediately after the abort and at 9 and 19 min after aborting the centrifugation. Venous blood samples were taken in 9 ml EDTA monovettes and immediately transferred into prechilled (4°C) aprotinin-EDTA tubes. The samples were cooled on crushed water ice until further processing. After spinning the aprotinin-EDTA blood samples in a 4°C temperature-controlled blood centrifuge (Heraeus Multifuge 1 S-R centrifuge) for 10 min at 3000 rpm, blood plasma was distributed in 0.5 ml portions in Eppendorf tubes and stored in a -80°C fridge until further processing at the Gravitational Physiology and Medicine Research unit, Medical University of Graz, Austria.

### Hormonal Analyses

After study completion, plasma galanin was measured using a commercially available radioimmunoassay (RIA) kit (Peninsula Laboratories International Inc., Belmont, CA, United States), after trifluoroacetic acid extraction. The eluate was stored at -80°C until the day assay was carried out. This RIA has an advantage that it does not cross-react with similar peptides, including insulin, secretin, substance P, or vasoactive intestinal peptide (VIP). The minimum detectable substance is 1.5 pmol l^-1^ (O’shea et al., 2015). The Shionogi Inc. (Osaka, Japan) immunoradiometric assay (IRMA) was used to measure plasma adrenomedullin concentrations of mature-type adrenomedullin (m-AM). Adrenomedullin is present in the plasma as biologically active, mature-type (amidated at the carboxy terminus), and a biologically intermediate type, which prior enzymatic amination is glycine-extended (O’shea et al., 2015). Using the one-step, two side IRMA process, this essay is able to detect m-AM quickly and reliably without cross-reacting with the intermediate type or similar peptides. The minimum detectable concentration of the substance is 0.5 pmol l^-1^ (O’shea et al., 2015).

### Data Processing and Statistical Analyses

Data and statistical analyses were performed with SPSS Statistics 21. Linear mixed effect (LME) models fitted by Restricted Maximum Likelihood estimation (REML) with gender, medical doctor, G_z_Feet_-load and position as fixed effects and subject ID as random effect were constructed in order to assess position, and G_z_-load and gender effects. The dependent variables were plasma concentration of adrenomedullin or galanin. Data are given as means and standard deviation (SD). The level of statistical significance was set to α = 0.05.

## Results

All subjects completed the study. Of the twenty subjects, nine subjects could complete the entire protocol in both sessions (finishers), whilst centrifugation had to be interrupted in eleven subjects, because of at least one of the pre-defined criteria for presyncope was fulfilled (non-finishers, Table 1). Non-finishers included five female and six male subjects; in five subjects, two presyncopal episodes occurred per visit (two times in female and three times in male subjects). Thirteen presyncopal events occurred after centrifugation with axis of rotation above the head whereas no presyncopal events appeared with axis of rotation positioned at the heart level. The medical doctor’s influence, which determined the subjects as non-finishers or finishers, was not revealed as significant (*p* > 0.05).

**Table 1:** Subject’s anthropometry data, mean ± SD.

		Number	Age (years)	Height (cm)	Weight (kg)	BMI (kg/m^2^)
Females	Finishers	5	26 ± 4	164 ± 5	58.7 ± 5.7	21.6 ± 1.6
	Non-finishers	5	25 ± 3	167 ± 3	68.0 ± 6.2	24.4 ± 2.8
Males	Finishers	4	25 ± 6	176 ± 4	74.5 ± 8.8	23.9 ± 1.8
	Non-finishers	6	27 ± 7	178 ± 5	73.8 ± 6.7	23.1 ± 1.2


*The table shows the measured anthropometry data of finisher and non-finisher subjects, separated by gender. BMI, body mass index; SD, standard deviation*.

### Galanin Response to Centrifugation

**FIGURE 2:**
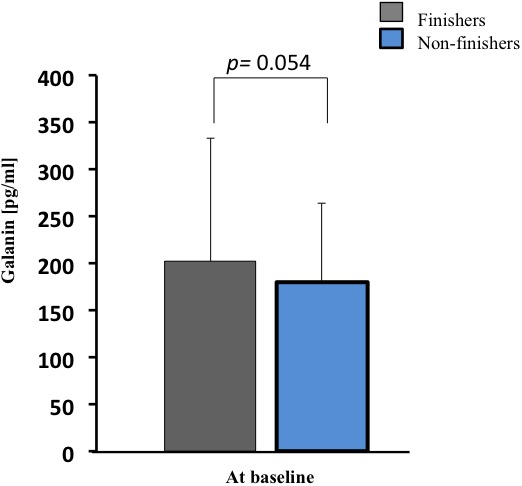
Galanin plasma concentration at baseline in finishers and non-finishers, prior to short arm human centrifugation (SAHC) application. Baseline hormone values were averaged from both baseline blood samples (see Figure 1A).

**FIGURE 3:**
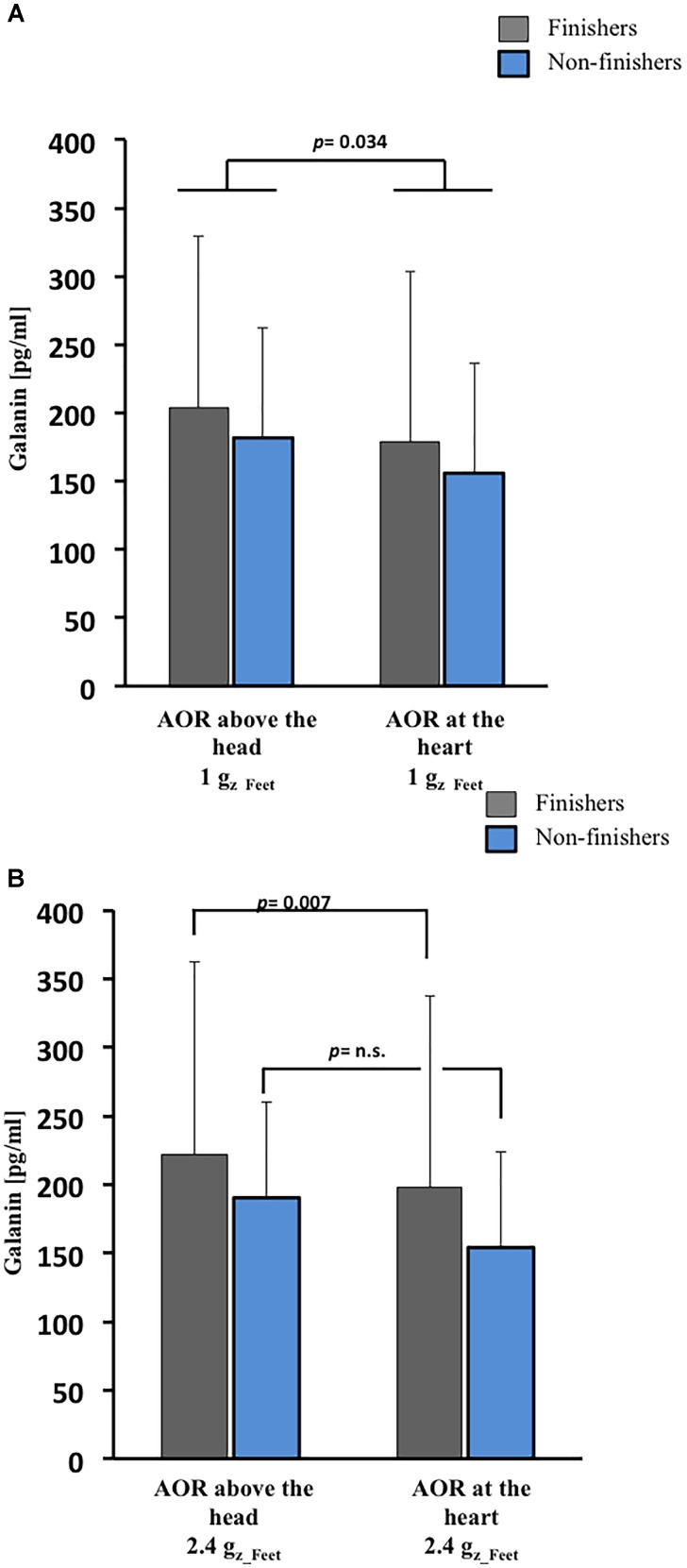
Galanin responses to SAHC based on varying position of AOR and g-level. **(A)** After 1 G_z___Feet_ centrifugation, galanin concentrations were dependent on g-gradient in both groups (*p* = 0.034). **(B)** After 2.4 G_z_Feet_ centrifugation, galanin concentrations were dependent on g-gradient (*p* = 0.007) in only the finishers. AOR, axis of rotation.

Finishers tended to have slightly greater galanin plasma concentrations than non-finishers at baseline (*p* = 0.054, Figure 2), but absolute galanin level changes were comparable between finishers and non-finishers following 1 G_z_Feet_ centrifugation. In both groups, galanin levels were lower after 1 G_z_Feet_ centrifugation with rotation axis placed at the heart compared with rotational axis placed above the head (*p* = 0.034, Figure 3A), suggesting an effect of gradient *ΔG_z_*. Indeed, galanin concentrations after rotation above the head with 1 G_z_Feet_ were almost comparable with baseline values in both groups.

Following exposure to centrifugation with 2.4 G_z_Feet_, galanin plasma concentration in finishers was significantly influenced by the applied gradient *ΔG_z_* (*p* = 0.007, Figure 3B). As with 1 G_z_Feet_ centrifugation, highest galanin levels were measured during 2.4 G_z_Feet_ centrifugation with axis of rotation positioned above the head in comparison with axis of rotation placed at the heart level.

### Adrenomedullin Response to Centrifugation

**FIGURE 4:**
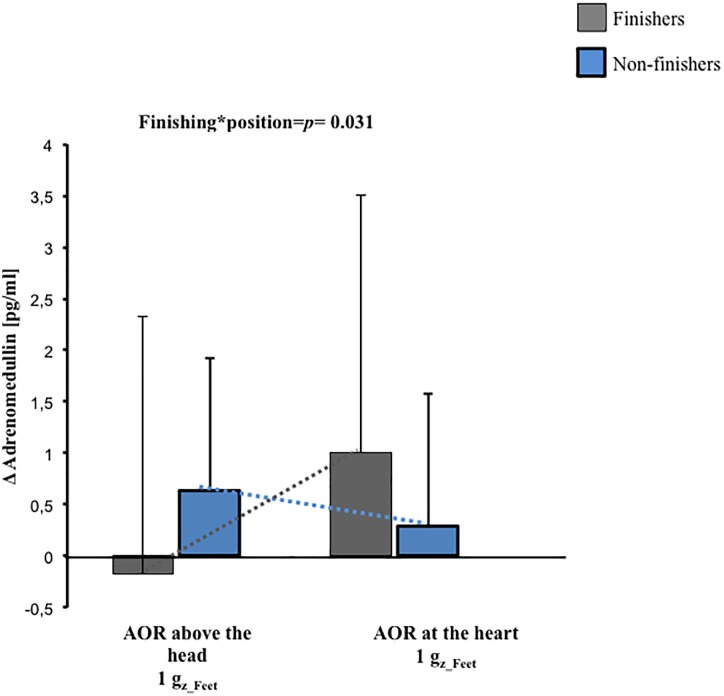
Pre-to-post change (Δ) in adrenomedullin concentrations and their interactions with g-gradient and orthostatic intolerance after 1 G_z_Feet_ centrifugation (*p* = 0.031). The data presented depict changes from the baseline. AOR, axis of rotation.

Baseline adrenomedullin plasma concentration were not different between finishers and non-finishers (finishers 5.5 ± 3.0 pg/ml, non-finishers 5.7 ± 2.7 pg/ml, *p* = 0.8). After 1 G_z_Feet_ centrifugation, a significant interaction between development of presyncopal symptoms and position of centrifugation was found (*p* = 0.031, Figure 4). Finishers had greatest adrenomedullin levels after centrifugation with axis of rotation located at the heart level, and non-finishers had greatest levels after centrifugation with axis of rotation placed above the head.

After exposure to centrifugation with 2.4 G_z_Feet_, neither the applied position nor the applied G_z_-level led to any differences in adrenomedullin levels (*p* ≥ 0.14).

### Effect of Gender on Hormonal Response

Galanin and adrenomedullin data showed no gender effect at baseline (*p* > 0.079 in all cases), neither after 1 G_z_Feet_ centrifugation with axis of rotation placed above the head nor at the heart level (*p* > 0.81 in all cases) nor after centrifugation with 2.4 G_z_Feet_ (*p* > 0.362 in all cases).

## Discussion

Short-arm human centrifugation with various locations of rotational axis represents a new method for studying the impact upon fluid shift to lower extremities and the accompanying endocrinological responses. As renin, aldosterone and vasopressin responses during AG application have been previously studied (Yang et al., 2011), in this study we assessed additional hormones that are altered during orthostatic loading and central hypovolemia.

### Galanin Changes During Centrifugation and at Presyncope

Elevated baseline levels of galanin appeared to predict orthostatic tolerance (*p* = 0.054) and seemed to support good orthostatic tolerance during 1 G_z_Feet_ SAHC (*p* = 0.034). Firstly, finishers showed higher galanin baseline levels as compared to non-finishers. In former studies, galanin was suggested as a stabilizer of cardiovascular responses during acute orthostatic challenge in HUT (Bondanelli et al., 2003). Although the pre-determined level of statistical significance of α = 0.05 was not reached in this current study, our results for galanin baseline concentration expand earlier findings. Our data suggest that galanin levels at baseline/during resting state and during SAHC application can predict the development of presyncopal signs and symptoms in an individual. Therefore, galanin should be considered, in combination with other clinical measurements (e.g., tilt table testing, Schellong test) (Stewart et al., 2017) as a marker for predicting presyncope during central hypovolemia induced by SAHC.

Secondly, we observed an influence by SAHC on galanin plasma concentrations. Existing literature is inconclusive regarding the role of galanin in orthostatic intolerance: researchers have reported increased galanin levels at the point of presyncope and a high galanin level association with orthostatic intolerance (Hinghofer-Szalkay et al., 2011; O’shea et al., 2015) while others have reported that in patients with HUT-induced syncope galanin does not change either preceding – or during – loss of consciousness (Bondanelli et al., 2003). The data reported from Bondanelli et al. (2003) suggest that endogenous galanin plays a key role in the adaptive responses to acute orthostatic challenge and prevents syncope in susceptible persons. In agreement with previously published studies, during central hypovolemia created by 1 G_z_Feet_ SAHC, increased plasma levels of galanin were seen.

Thirdly, galanin levels in finishers after centrifugation at 2.4 G_z_Feet_ were significantly increased in comparison to baseline and 1 G_z_Feet_ centrifugation values. Independent of the used position, SAHC with such high g-levels represents a high stress level for the cardiovascular system. Our galanin results after 2.4 G_z_Feet_ may reflect a high level of sympathetic activity, which is in accordance with galanin’s role in preventing presyncope, which was reported in previous studies (Bondanelli et al., 2003). In comparison to finishers, in non-finishers galanin levels were not increased during SAHC; this in turn, could have contributed to their development of presyncope.

Our results appear to disclaim our hypothesis related to plasma galanin levels before SAHC application, after 1 G_z_Feet_ and after 2.4 G_z_Feet_ SAHC. In contrast to former studies, our results are indicative of galanin’s stabilizing effect for coping with graded central hypovolemia when compared to non-finishers during SAHC exposure.

### Adrenomedullin Changes During Centrifugation and at Presyncope

No significant differences in adrenomedullin were seen at baseline between finishers and non-finishers. It has been reported that adrenomedullin acts as an effective paracrine vasodilator showing a specific dose-dependent releasing during orthostatic stimulus (Kitamura et al., 1993; Rossler et al., 1999; Eto, 2001; Bondanelli et al., 2003). Our results are in accordance with previous studies, which reported no difference before application of the orthostatic stimulus between finishers and non-finishers (Kitamura et al., 1993; Gajek et al., 2004).

Furthermore, we observed a significant interaction in adrenomedullin concentration changes between finishers and non-finishers after 1 G_z_Feet_ centrifugation dependent on the applied position. Finishing the protocol without developing syncope was related to stable adrenomedullin plasma levels after centrifugation with axis of rotation placed above the head in comparison with increased adrenomedullin levels in non-finishers at the same position. Our results are in accordance with recent studies, which reported that orthostatic intolerance (assessed using HUT + graded LBNP until presyncope) following 21-day of bedrest was associated with increased adrenomedullin levels (O’shea et al., 2015). Inversely, in non-finishers, adrenomedullin plasma levels were comparable to baseline levels and increased in orthostatic tolerant subjects after centrifugation with axis of rotation placed at heart level. Our results suggest that adrenomedullin plasma concentrations after SAHC are dependent on the degree of central hypovolemia and ability to cope with it. Further, adrenomedullin plasma changes after SAHC may not be always comparable to those obtained using other orthostatic loading stressors (Gajek et al., 2004; O’shea et al., 2015). Moreover, our findings of adrenomedullin concentrations after exposure to centrifugation at 2.4 G_z_Feet_ suggest that it might have no relevance to coping responses during such exposures. Based on previous evidence, we speculate that factors other than local distribution of adrenomedullin may be involved in coping with central hypovolemia caused by 2.4 G_z_Feet_ SAHC (Nishikimi et al., 2001; Gajek et al., 2004; Hinghofer-Szalkay et al., 2011).

In summary, following 1g G_z_Feet_ SAHC with axis of rotation placed above the head (that is, which induces a high degree of central hypovolemia) adrenomedullin levels increased in non-finishers. However, following SAHC with axis of rotation placed at the heart level (that is, which induces a lower central hypovolemia), adrenomedullin plasma concentrations in non-finishers were reduced in comparison to finishers. Following 2.4g G_z_Feet_ SAHC, adrenomedullin plasma levels showed no significant different between non-finishers and finishers. The adrenomedullin data obtained in our study only partially support our proposed hypothesis.

### Gender Differences in Hormonal Responses

Interestingly, neither the number of presyncopal episodes nor the hormonal concentrations of the subjects during our centrifuge protocol showed any statistically significant gender difference. This contrasts with a former study, which suggested gender differences in hormonal concentrations during presyncope in galanin (Hinghofer-Szalkay et al., 2006). The differences in the results might be explained by the stress of SAHC itself, which represents a different orthostatic stimulus in comparison to HUT and LBNP used in the other study. Due to limited data available in gender specific responses to central hypovolemia (Evans et al., 2018), future studies should focus on gender differences (Clement et al., 2016).

### Limitations

As the time required to complete a single centrifuge run was long, the subjects were not spun at the same time of the day. Therefore, effects of circadian rhythms on the both of these hormones could not be accounted for. However, studies that have previously examined adrenomedullin responses have not reported any effects of circadian rhythms on adrenomedullin levels (Nishikimi et al., 2001; Nishikimi, 2005; Clement et al., 2016). Similarly, we could not find any study that examined the effects of circadian rhythms on galanin.

Furthermore, the total sample size (*n* = 20) is probably too small to detect subtle gender differences. However, this study is the first to report involvement of at least one the two under-studied hormones, namely galanin and adrenomedullin, during graded central hypovolemia induced by G_z_ loading. The hormonal data obtained in this pilot study can, therefore, serve as a basis for sample size calculations in future epidemiological studies.

## Conclusion and Further Directions

We assessed whether galanin and adrenomedullin plasma concentration changes during central hypovolemia observed in other studies are transferable to SAHC, especially with varying gravitational loading and varying positions of axis of rotation of the centrifuge. We observed that galanin plasma levels are not only important during orthostatic challenge but could also predict development of presyncope in subjects undergoing centrifugation. Therefore, we recommend in future studies the measurement of baseline galanin levels – in combination with other clinical tools (e.g., questionnaire, tilt-test or Schellong-test) – to confirm galanin’s ability to predict development of presyncope in subjects. Furthermore, as falls occur during changes in posture, especially in older persons (Goswami et al., 2018, 2019; Trozic et al., 2019), our data could have clinical relevance. Galanin and adrenomedullin could act as biomarkers for predicting which older persons can potentially develop orthostatic intolerance.

In addition, it appears that SAHC offers a novel approach to explore the effect of volume shift upon hormonal control of the cardiovascular system. It should also be noted that the gradient acceleration field in SAHC will engender a hydrostatic pressure profile that increases quadratically with the distance from the center of rotation, which contrasts with the linear profile in constant 1g-fields. Based on our novel results, the gradient of acceleration *ΔG_z_* appears to be optimal for application of centrifugal acceleration.

## Author Contributions

JW conducted the study, performed the statistical analysis and interpreted the data for the work, and drafted the work and revised it critically for important intellectual content. CL and EM conceived and designed the study and conducted the study. BJ performed the statistical analysis. JoRe, AR, and BB drafted the work and revised it critically for important intellectual content. JR and NG conceived and designed the study, interpreted the data and helped in editing the written manuscript.

## Conflict of Interest Statement

Parts of this manuscript were presented at the “1st Human Physiology Workshop” in Cologne, Germany, December 10, 2016 and at the “16. Tag der Forschung der Universität Duisburg-Essen” in Essen, Germany, November 17, 2017 by Julia Stroetges and during the “Aerospace Conference 2016” in Montana, United States, March 5–12, 2016 by CL (Laing et al., 2016). The remaining authors declare that the research was conducted in the absence of any commercial or financial relationships that could be construed as a potential conflict of interest. The reviewer JE declared a past co-authorship with one of the authors NG to the handling Editor.

## Abbreviations

HUT: head-up tilting
LBNP: lower body negative pressure
SAHC: short-arm human centrifugation
